# *Candida albicans* Infection of *Caenorhabditis elegans* Induces Antifungal Immune Defenses

**DOI:** 10.1371/journal.ppat.1002074

**Published:** 2011-06-23

**Authors:** Read Pukkila-Worley, Frederick M. Ausubel, Eleftherios Mylonakis

**Affiliations:** 1 Division of Infectious Diseases, Massachusetts General Hospital, Boston, Massachusetts, United States of America; 2 Department of Molecular Biology, Massachusetts General Hospital, Boston, Massachusetts, United States of America; 3 Department of Genetics, Harvard Medical School, Boston, Massachusetts, United States of America; 4 Harvard Medical School, Boston, Massachusetts, United States of America; University of Massachusetts Medical School, United States of America

## Abstract

*Candida albicans* yeast cells are found in the intestine of most humans, yet this opportunist can invade host tissues and cause life-threatening infections in susceptible individuals. To better understand the host factors that underlie susceptibility to candidiasis, we developed a new model to study antifungal innate immunity. We demonstrate that the yeast form of *C. albicans* establishes an intestinal infection in *Caenorhabditis elegans*, whereas heat-killed yeast are avirulent. Genome-wide, transcription-profiling analysis of *C. elegans* infected with *C. albicans* yeast showed that exposure to *C. albicans* stimulated a rapid host response involving 313 genes (124 upregulated and 189 downregulated, ∼1.6% of the genome) many of which encode antimicrobial, secreted or detoxification proteins. Interestingly, the host genes affected by *C. albicans* exposure overlapped only to a small extent with the distinct transcriptional responses to the pathogenic bacteria *Pseudomonas aeruginosa* or *Staphylococcus aureus*, indicating that there is a high degree of immune specificity toward different bacterial species and *C. albicans*. Furthermore, genes induced by *P. aeruginosa* and *S. aureus* were strongly over-represented among the genes downregulated during *C. albicans* infection, suggesting that in response to fungal pathogens, nematodes selectively repress the transcription of antibacterial immune effectors. A similar phenomenon is well known in the plant immune response, but has not been described previously in metazoans. Finally, 56% of the genes induced by live *C. albicans* were also upregulated by heat-killed yeast. These data suggest that a large part of the transcriptional response to *C. albicans* is mediated through “pattern recognition,” an ancient immune surveillance mechanism able to detect conserved microbial molecules (so-called pathogen-associated molecular patterns or PAMPs). This study provides new information on the evolution and regulation of the innate immune response to divergent pathogens and demonstrates that nematodes selectively mount specific antifungal defenses at the expense of antibacterial responses.

## Author Summary

Despite being a part of the normal flora of healthy individuals, *Candida albicans* is the most common fungal pathogen of humans and can cause infections that are associated with staggeringly high mortality rates. Here we devise a model for the study of the host immune response to *C. albicans* infection using the nematode *C. elegans*. We found that infection with the yeast form of *C. albicans* induces rapid and robust transcriptional changes in *C. elegans*. Analyses of these differentially regulated genes indicate that the nematode mounts antifungal defenses that are remarkably distinct from the host responses to pathogenic bacteria and that the nematode recognizes components possessed by heat-killed *C. albicans* to initiate this response. Interestingly, during infection with a pathogenic fungus, the nematode downregulates antibacterial immune response genes, which may reflect an evolutionary tradeoff between bacterial and fungal defense.

## Introduction

*Candida albicans* is a remarkably successful and versatile human pathogen that is found on the skin and mucosal surfaces of virtually all humans. Under most circumstances, *C. albicans* is a harmless commensal [1]. However, this opportunist can invade host tissues and cause life-threatening infections when the immune system is weakened (e.g. from critical illness) and competing bacterial flora are eliminated (e.g. from broad-spectrum antibiotic use). Accordingly, invasive candidiasis is particularly common in intensive care units where mortality rates reach 45–49% [2]–[4]. Antecedent colonization of mucosal surfaces with *C. albicans* can also lead to debilitating superficial infections in otherwise normal hosts. Approximately 75% of all women, for example, will have one episode of *Candida* vaginitis in their lifetime, with half having at least one recurrence [5].

*C. albicans* can grow vegetatively as yeast or hyphae, and each form contributes to pathogenesis [6]–[8]. *C. albicans* yeast cells colonize mucosal surfaces and facilitate dissemination of the organism through the blood stream [9]–[11]. Hyphae, by contrast, are important for host invasion and tissue destruction [1], [8], [11], [12]. The factors that influence these diverse growth patterns during infection are poorly understood, but it is clear that innate immune mechanisms in mammalian epithelial cells normally prevent *C. albicans* from becoming a pathogen [13]–[15]. Recently, genetic analyses of two human families whose members suffered from recurrent or chronic candidiasis on mucosal surfaces identified causative mutations in the innate immune regulators dectin-1 [16] and *CARD9* [17]. Dectin-1 is a pattern-recognition receptor important for macrophage phagocytosis of fungi. Interestingly, this protein interacts differently with the *C. albicans* growth forms. Cell wall components exposed in the bud scar of *C. albicans* yeast (so-called pathogen-associated molecular patterns or PAMPs) potently stimulate dectin-1, but hyphae are relatively shielded from innate immune detection, which likely contributes to the ability of *C. albicans* to establish infection [13], [15], [18]. Furthermore, a recent study found that the p38 MAP kinase, a central regulator of mammalian immunity, receives biphasic inputs from *C. albicans* that are dependent on the morphologic form of the organism and the local fungal burden [14]. These data suggest that the interplay between *C. albicans* and the mammalian innate immune system dictate the virulence potential of this specialized pathogen, yet relatively little is known about the molecular mechanisms underlying these interactions.

One approach to study evolutionarily conserved aspects of epithelial innate immunity and microbial virulence uses the invertebrate host *Caenorhabditis elegans* [19], [20]. In nature, nematodes encounter numerous threats from ingested pathogens, which have provided a strong selection pressure to evolve and maintain a sophisticated innate immune system in its intestinal epithelium [21]. Coordination of these defenses involves several highly-conserved elements that have mammalian orthologs [22]–[25]. Furthermore, *C. elegans* intestinal epithelial cells bear a striking resemblance to human intestinal cells [26] and because the nematode lacks both a circulatory system and cells dedicated to the immune response, the intestinal epithelium constitutes the primary line of defense for the nematode against ingested pathogens. Thus, it is possible to conduct analyses of innate immune mechanisms in a physiologically-relevant, genetically-tractable system.

Much of the characterization of nematode immunity has used nosocomial bacterial pathogens [27]–[30], particularly *Pseudomonas aeruginosa* [22], [31], [32], but to date, the immune response directed toward a medically-important, fungal pathogen has not been defined. Here, we extend our previously-validated system for the study of hyphal-mediated *C. albicans* virulence in the nematode [33] to examine *C. albicans* yeast. Our goal was to use studies of *C. elegans-C. albicans* interactions to identify novel, conserved features of metazoan innate immunity. We found that the responses to bacterial and fungal pathogens are remarkably distinct. Many of the immune response effectors that are upregulated by either *P. aeruginosa* or *S. aureus* are downregulated by infection with *C. albicans* yeast. We also found that slightly more than half of the immune response genes activated by infection with live *C. albicans* are also upregulated by heat-killed *C. albicans*. Our data indicate that the *C. elegans* immune response to *C. albicans* most likely involves detection of conserved surface-associated molecular pattern molecules, as well as detection of *C. albicans* virulence-related factors.

## Results

### The Yeast Form of *C. albicans* is Pathogenic to *C. elegans*

**Figure 1:**
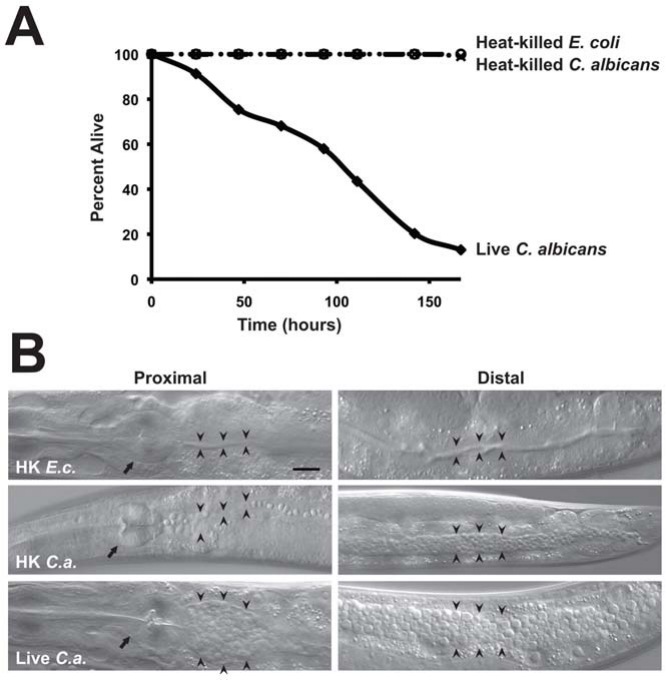
*C. albicans* yeast can kill *C. elegans*. (A) Live *C. albicans* (closed diamonds) were pathogenic to nematodes on solid media, whereas heat-killed *C. albicans* (open circles) and *E. coli* (crosses) were not (*P*<0.001). The graph presents the average of three plates per strain, each with 30 to 40 animals per plate. Data are representative of two biological replicates. (B) Images of *C. elegans* animals exposed to heat-killed *E. coli* (HK *E.c.*), heat-killed *C. albicans* (HK *C.a.*) or live *C. albicans* (live *C.a.*) for 16 hours at 25°C are shown. Images of the proximal (left) and distal (right) intestine were obtained using Nomarski optics. Both live and heat-killed *C. albicans* accumulated within the intestine, but only live *C. albicans* caused marked distention of the proximal intestine. Arrows point to the pharyngeal grinder and arrowheads outline the lumen of the intestine. The scale bar represents 20 µm.

**Figure 2:**
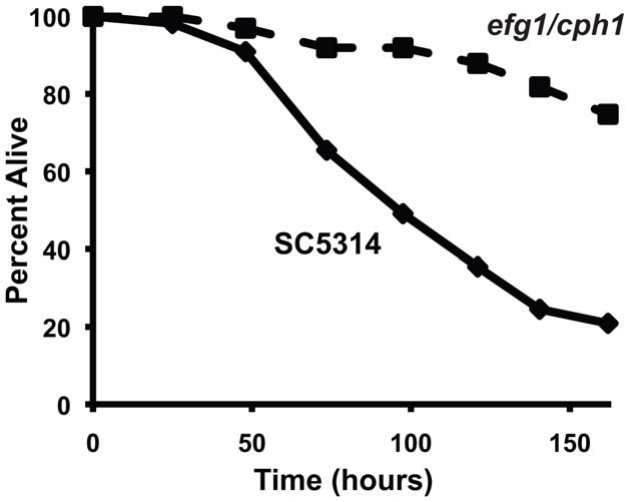
A *C. albicans* double mutant strain that is attenuated for pathogenicity in mammals is also unable to efficiently kill *C. elegans*. The *C. albicans efg1Δ/efg1Δ cph1Δ/cph1Δ* double mutant strain (*efg1/cph1*) exhibited a reduced ability to kill *C. elegans* compared to its isogenic wild-type parent strain SC5314 (*P*<0.001). The graph presents the average of three plates per strain, each with 30 to 40 animals per plate. Data are representative of two biological replicates.

To examine interactions between *C. albicans* and the innate immune system, we established a novel system using the model host *C. elegans*. In a previous study, we found that *C. albicans* hyphae can kill *C. elegans* in a manner that models key aspects of mammalian pathogenesis [20], [33]. In that assay, yeast cells were ingested by nematodes on solid medium and, after transfer to liquid medium, worms died with true hyphae piercing through their bodies. During these experiments, we noted that when infected worms were maintained on solid media, rather than transferred to liquid media, the *C. albicans* yeast form caused pathogenic distention of the nematode intestine and premature death of the worms. Thus, we hypothesized that *C. albicans* yeast, the form commonly found in the mammalian intestine [13], [15], [18], also contain virulence determinants that allow infection of *C. elegans*. We therefore developed an assay that is conducted exclusively on solid media and allows the direct study of yeast-mediated pathogenesis of the nematode. As shown in Figure 1, the yeast form of the *C. albicans* laboratory reference strain DAY185 infected and killed *C. elegans*. Heat-killed *C. albicans* yeast cells were not pathogenic to the nematode (Figure 1A) and caused less distention of the nematode intestine compared to that seen following exposure to live *C. albicans* (Figure 1B). We found that the *C. albicans* clinical isolate SC5314 was also able to establish a lethal infection in nematodes (Figure 2). Furthermore, the *C. albicans efg1Δ/efg1Δ cph1Δ/cph1Δ* double mutant strain [8], which is attenuated for virulence in mammals, was also unable to efficiently kill *C. elegans* in this assay (Figure 2). Like its isogenic wild-type parent strain, virulence-attenuated *C. albicans* yeast enter the nematode intestine during the infection assay (data not shown), suggesting that non-specific occlusion of the intestine with yeast is not the mechanism of *C. albicans*-mediated worm killing. In addition, we found that *C. albicans* killed sterile *C. elegans fer-15(b26);fem-1(hc17)* animals (data not shown) and wild-type worms in the presence of 5-fluoro-2′-deoxyuridine (FUDR), a compound that prevents progeny from hatching (Figure 1A). These results suggest that killing of nematodes by *C. albicans* yeast in the *C. elegans* model involves virulence determinants intrinsic to live fungi and not a “matricidal effect” from premature hatching of embryos inside animals, a previously described, non-specific consequence of pathogen stress in wild-type worms [26], [31], [32], [34]. In summary, these data demonstrate that *C. albicans* yeast are pathogenic to the nematode and establish a second assay, which together with the liquid-media system [33], permit separate *in vivo* analyses of *C. albicans* growth states.

### *C. albicans* Infection Induces a Rapid Host Response that Involves Antimicrobial, Secreted and Detoxification Genes

**Figure 3:**
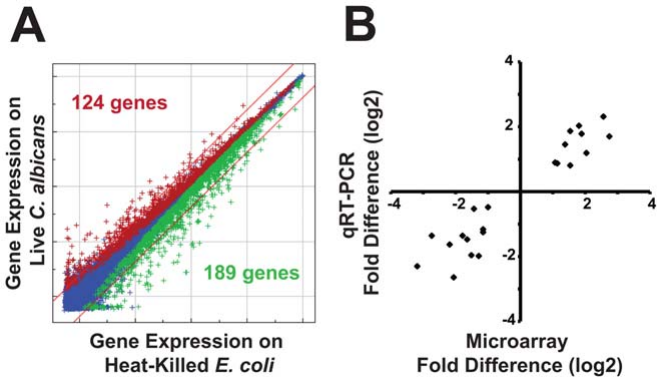
Infection with *C. albicans* yeast induces a rapid host response. (A) *C. elegans* genes that were differentially regulated in *C. albicans*-exposed versus heat-killed *E. coli*-exposed young adult animals at 4 hours after infection are depicted on a genome-wide intensity plot of 22,548 sequences. Genes colored red were upregulated by *C. albicans* (*P*<0.01), those colored green were downregulated (*P*<0.01) and those colored blue were unchanged. Diagonal lines represent 2-fold change and the numbers of genes differentially regulated greater than 2-fold are indicated (*P*<0.01)(124 genes were upregulated and 189 genes were downregulated). (B) qRT-PCR was used to confirm the results of the microarray analysis. 11 genes with varying degrees of differential regulation were selected and studied under each condition in which they were differentially regulated in the microarray analysis (see Table S2 for gene identities). Correlation of microarray and qRT-PCR data was determined by plotting the average fold difference observed in the microarray analysis (three biological replicates) versus the average fold difference for the same gene obtained by qRT-PCR (three biological replicates). Linear regression analysis revealed strong correlation between the datasets (R^2^ of 0.90).

Previous studies have shown that *C. elegans* mounts a rapid and specific immune response toward pathogenic bacteria [32], [35], [36]; however, it is not known how the nematode defends itself against an intestinal fungal pathogen. We therefore used transcriptome profiles of nematodes during an infection with *C. albicans* yeast to define the antifungal immune response genes in the nematode. We compared gene expression of animals exposed to *C. albicans* for four hours with control worms fed the non-pathogenic food source, heat-killed *E. coli* OP50. The short exposure time maximized the yield for transcriptional changes associated with pathogen detection, rather than gene expression changes associated with intestinal damage [36]. It was necessary to use heat-killed *E. coli* for these experiments because live *E. coli* were previously shown to be pathogenic to the nematode on *C. albicans* growth media (brain heart infusion agar) [37]. We found that *C. elegans* coordinates a rapid and robust transcriptional response to *C. albicans* that involves approximately 1.6% of the nematode genome (Figure 3). 124 genes were upregulated two-fold or greater in response to *C. albicans* compared to heat-killed *E. coli* and 189 genes were downregulated at least two-fold (*P*<0.01) (Figure 3A and Table S1A). For technical confirmation of the microarray experiment, we selected 11 genes that showed varying degrees of differential regulation and tested their expression by quantitative real-time polymerase chain reaction (qRT-PCR) under each microarray condition (Figure 3B and Table S2). Plotting the fold difference observed in the transcriptome profiles versus the value obtained by qRT-PCR from the three biological replicates used for the microarray analysis yielded an R^2^ of 0.90 (Figure 3B), which indicates tight correlation between these datasets and is a result that compares favorably with similar analyses of other microarray experiments [38]. We also tested three additional biological replicates and found similar fold changes between the microarray and qRT-PCR analyses in 10 of the 11 genes (Table S2), a correlation rate that is consistent with other microarray analyses of pathogen response genes in the nematode [34]. As a third means to confirm the results of our microarray, we compared the expression of 4 upregulated and 4 downregulated genes in wild-type *C. elegans* animals infected with a different *C. albicans* strain than used for the microarray analysis. We exposed animals to the *C. albicans* clinical isolate SC5314, a strain that is also virulent toward *C. elegans* (Figure 2), and found similar transcriptional changes between *C. albicans* SC5314 and DAY185-exposed animals for all 8 genes tested (Table S2). These data suggest that the *C. albicans*-induced transcriptional changes observed in our microarray analysis are not specific to a particular yeast strain.

Examination of the genes induced by *C. albicans* in the microarray analysis reveals the footprint of an immune response toward a pathogenic fungus (Table 1). *C. albicans* infection results in the elaboration of at least seven putative antimicrobial peptides, which are postulated to have antifungal activity *in vivo*. One of these genes, *abf-2*, was previously shown to have *in vitro* activity against the pathogenic fungus *Candida krusei* [39]. Three genes in this group (*fipr-22/23* and two caenacin genes, *cnc-4* and *cnc-7*) are antifungal immune effectors induced by the nematode following exposure to *Drechmeria coniospora*, an environmental fungal pathogen, which causes a localized infection of the nematode cuticle [40], [41]. *fipr-22* and *fipr-23* have nearly identical DNA sequences and thus, it is not possible for a probe set to distinguish between these genes. Two chitinase genes (*cht-1* and *T19H5.1*) were also strongly induced by *C. albicans*. These enzymes are secreted by metazoans and are thought to defend against chitin-containing microorganisms such as *C. albicans* and other pathogenic fungi [42], [43]. In addition, *thn-1*, a gene that is postulated to have direct antimicrobial activity and is a homolog of the thaumatin family of plant antifungals [35], [44], was induced 2.5-fold during infection with *C. albicans*.

**Table 1:** The *C. elegans* transcriptional response to *C. albicans* infection involves antimicrobial, detoxification and other pathogen-response genes.

Sequence name	Gene name	Sequence description	Fold Change	*P* value	Induced by heat-killed *C. albicans*	Presumptive function	Signal sequence [85]	Gut Expression
*F44E5.4,F44E5.5*		Hsp70 family of heat shock proteins	14.0	0.0001	-	Pathogen response [86]–[88]	-	Yes [82]
*F52F10.3*	*oac-31*	Predicted acyltransferase	10.1	3.2×10^−7^	Yes	Detoxification [32], [34], [35], [89]	Yes	Yes [82]
*M01H9.1*	*trx-3*	Thioredoxin, nucleoredoxin	9.6	3.4×10^−11^	Yes	Detoxification [32], [89]	-	Yes [81]
*ZK550.2*		Predicted transporter/transmembrane protein	8.4	0.00006	Yes		-	Yes [81]
*C37A5.2,C37A5.4*	*fipr-22, fipr-23*	Presumptive antimicrobial peptide	6.7	0.002	-	Antimicrobial [40]	Yes	Yes [82]
*C50F2.10*	*abf-2*	Antimicrobial peptide	5.9	4.2×10^−14^	Yes	Antimicrobial [39]	Yes	Yes [39]
*T07G12.5*		Xanthine/uracil/vitamin C transporter, Permease	5.4	0.00008	Yes	Detoxification [32], [89]	Yes	- [82]
*C54F6.14*	*ftn-1*	Ferritin heavy chain homolog	4.9	1.2×10^−18^	Yes	Stress response [90]	-	Yes [90]
*T19H5.1*		Chitinase	4.7	0.002	-	Antimicrobial [42], [43]	Yes	
*C01G6.7*	*acs-7*	Acyl-CoA synthetase	4.5	1.2×10^−17^	Yes	Pathogen response [32]	-	Yes [82]
*Y60C6A.1*			4.4	1.0×10^−7^	Yes	Pathogen response [32]	Yes	
*R09B5.9*	*cnc-4*	Caenacin antimicrobial peptide	4.1	1.9×10^−11^	Yes	Antimicrobial [41]	Yes	Yes [82]
*T09B9.2*		Permease	4.1	0.01	-	Detoxification [34]	Yes	
*Y46H3A.4*		Predicted lipase	4.0	9.6×10^−6^	Yes	Antimicrobial [34]	-	
*T21C9.8*		Transthyretin-like family	4.0	0.001	Yes	Pathogen response [32]	Yes	
*T06D8.1*		Domain of unknown function	3.9	5.6×10^−24^	Yes		Yes	
*Y38E10A.15*	*nspe-7*	Nematode specific peptide family	3.6	0.002	-		Yes	
*F58E10.7*			3.6	2.9×10^−15^	Yes		Yes	
*C25H3.10*		Cyclin-like F-box domain	3.6	4.6×10^−17^	-	Pathogen response [32]	-	
*F35E12.5*		CUB-like domain	3.5	4.9×10^−10^	Yes	Pathogen response [29], [32], [35]	Yes	Yes [29]
*C04F6.3*	*cht-1*	Chitinase	3.4	1.5×10^−12^	Yes	Antimicrobial [42], [43]	Yes	
*R05H10.1*			3.3	2.6×10^−6^	-		-	Yes [82]
*C04F5.7*	*ugt-63*	UDP-glucuronosyl and UDP-glucosyl transferase	3.2	0.005	-	Detoxification [32], [89]	Yes	
*T16G1.4*		Domain of unknown function	3.2	6.0×10^−6^	Yes		-	
*F58H1.7*		Low density lipoprotein-receptor	3.1	0.001	-		Yes	
*C33D9.1*	*exc-5*	Guanine nucleotide exchange factor for cdc-42	3.1	0.007	Yes		-	
*F13E9.11*			3.1	2.0×10^−6^	Yes	Pathogen response [32], [35]	-	
*F49E11.10*	*scl-2*	SCP/TAPS domain-containing secretory protein	3.1	1.8×10^−31^	Yes	Pathogen response [32], [35]	Yes	Yes [82]
*C04A11.3*	*gck-4*	Ste20-like serine/threonine protein kinase	3.0	0.0004	-	Pathogen response [91]	-	Yes [81]
*F18C5.10*			3.0	1.3×10^−15^	-		-	
*Y41D4B.16*		Domain of unknown function	3.0	0.0001	Yes	Pathogen response [29], [32]	Yes	
*Y80D3A.7*	*ptr-22*	Sterol sensing domain protein	3.0	0.00001	-		Yes	
*Y38E10A.16*	*nspe-5*		3.0	0.01	-		Yes	

Genes upregulated 3-fold or more by *C. albicans* compared to heat-killed *E. coli* are presented along with their associated *P* values. Genes that were also induced by heat-killed *C. albicans* versus heat-killed *E. coli* (*P*<0.01) are indicated. The cited references were used to determine the presumptive function of the genes and whether the gene is expressed in the gut. The presence of a signal sequence suggests that the gene product is secreted and was determined using SignalP 3.0 [85]. “-” means an answer of ‘No’ and a blank cell in the table indicates that information was not available. The Affymetrix probes for *F44E5.4/5* and *C37A5.2/4* could not distinguish between the individual genes owing to sequence similarity.

**Figure 4:**
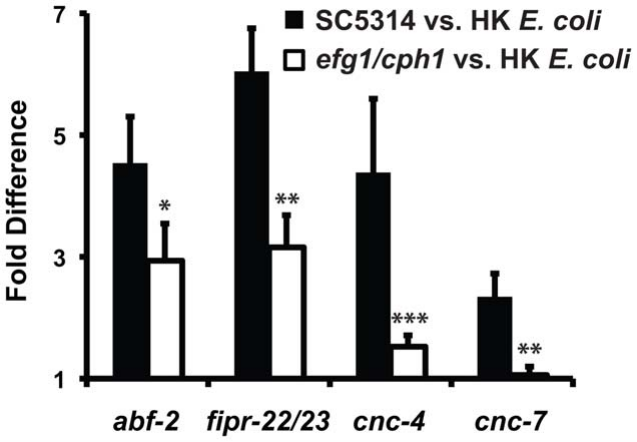
The virulence of the infecting *C. albicans* strain affects the induction of putative antifungal immune effectors. The induction of *abf-2*, *fipr-22/23*, *cnc-4* and *cnc-7* is reduced in wild-type *C. elegans* animals during infection with the virulence-attenuated *C. albicans efg1Δ/efg1Δ cph1Δ/cph1Δ* double mutant strain [vs. heat-killed (HK) *E. coli*] compared to its isogenic wild-type parent strain SC5314 (vs. heat-killed *E. coli*). Data are presented as the average of three biological replicates, each conducted in duplicate and normalized to a control gene with error bars representing SEM. *P = 0.06, **P<0.01 and ***P<0.025 for the comparison of gene induction on SC5314 versus *efg1Δ/efg1Δ cph1Δ/cph1Δ*.

Using gene expression analyses, we characterized further the expression pattern of four putative antifungal immune effectors upregulated during *C. albicans* infection (*abf-2*, *fipr-22/23, cnc-4* and *cnc-7*). We exposed wild-type nematodes to the *C. albicans efg1Δ/efg1Δ cph1Δ/cph1Δ* double mutant, a strain that is attenuated for virulence in *C. elegans* (Figure 2) and mammals [8], and found that the induction of *abf-2*, *fipr-22/23*, *cnc-4* and *cnc-7* was reduced compared to its isogenic parent strain *C. albicans* SC5314 (*P*<0.01 for *fipr-22/23* and *cnc-7*, *P* = 0.06 for *abf-2*, *P*<0.025 for *cnc-4*)(Figure 4). These data suggest that the nematode modulates the expression levels of antifungal immune effectors in response to some aspect of *C. albicans* virulence, although this yeast may be recognized differently by the nematode innate immune system owing to pleotropic effects of the genetic lesions in this mutant strain. We also found that the induction levels of these four genes appear to be dynamic during infection. Twelve hours after exposure to *C. albicans*, the expression of *abf-2* increases significantly, *fipr-22/23* is unchanged and *cnc-4* and *cnc-7* is reduced (Figure S1).

Among the most highly upregulated *C. albicans* defense genes (Table 1), we also identified a preponderance of genes encoding secreted proteins, intestinally-expressed proteins and proteins that may function as detoxifying enzymes. Similar types of genes are induced following infection with pathogenic bacteria [32], [34]. As discussed in more detail below, we also found that some of the *C. albicans*-induced genes were involved in the nematode transcriptional response to bacterial pathogens (Table 1), suggesting that *C. albicans* and pathogenic bacteria induce a set of common immune response effectors. Although it is possible that the effects of nematode starvation are also reflected in the transcription profiling data as a potential consequence of *C. albicans* being comparatively non-nutritious relative to heat-killed *E. coli*, this seems less likely since zero of the eighteen previously-identified, fasting-affected genes [45] were differentially expressed in the dataset. Taken together, these data suggest that the microarray analysis captured the early defense response mounted by *C. elegans* toward an ingested fungal pathogen.

### The Conserved PMK-1/p38 MAP Kinase Mediates Resistance to *C. albicans* Infection

**Figure 5:**
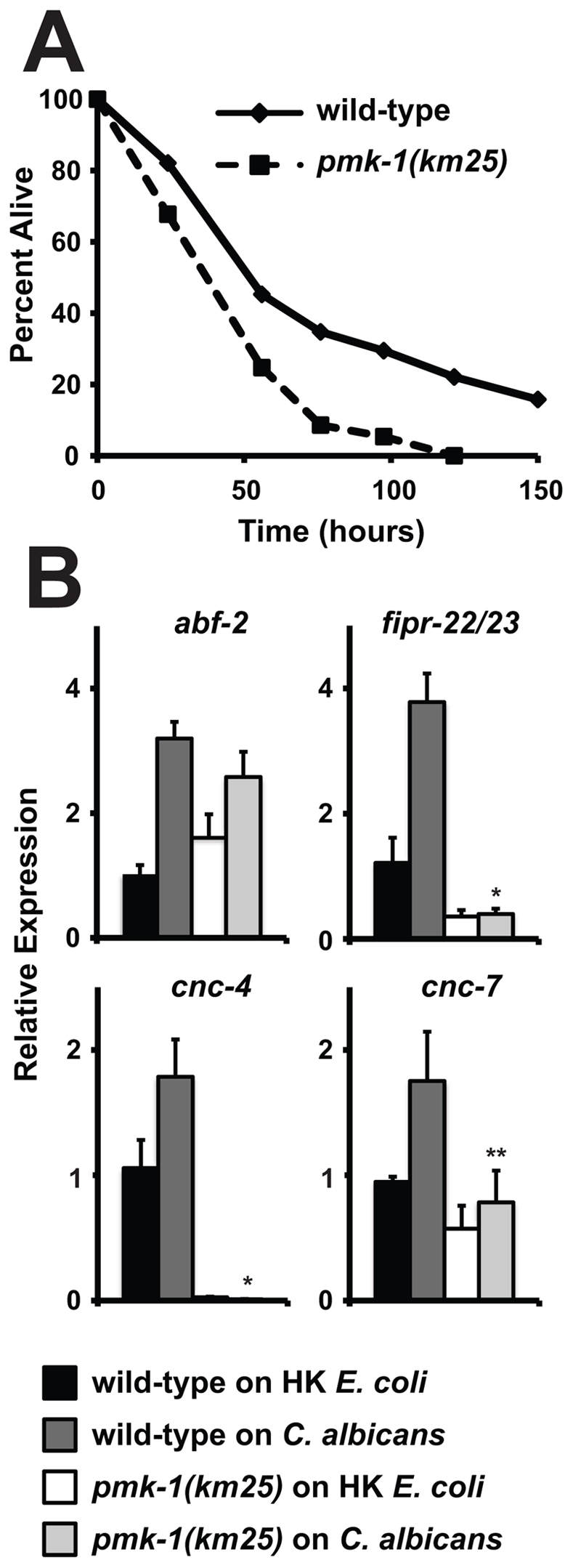
The p38 MAP Kinase PMK-1 is Required for the response to *C. albicans* infection. (A) A *C. albicans* infection assay with wild-type (N2) and *pmk-1(km25)* animals shows that *pmk-1(km25)* mutants were more susceptible to *C. albicans* infection (*P*<0.01). Each time point represents the average of three plates per strain, each with 30 to 40 animals per plate. Data are representative of two independent experiments. (B) N2 and *pmk-1(km25)* young adult animals were exposed to the indicated food source and the indicated genes were studied using qRT-PCR (HK equals heat-killed). Expression is relative to N2 on heat-killed *E. coli* and the data are presented as the average of three biological replicates each normalized to a control gene with error bars representing SEM. *P<0.001 and **P equals 0.05 for the comparison of relative expression of the indicated gene in wild-type animals on *C. albicans* versus *pmk-1(km25)* animals on *C. albicans*.

Genetic, biochemical and molecular analyses have identified a requirement for the PMK-1 mitogen-activated protein (MAP) kinase, orthologous to the mammalian p38 MAPK, in *C. elegans* immunity [22], [29], [46]–[48]. PMK-1 is a central regulator of nematode defenses [32] that acts cell autonomously both in the intestine to control resistance toward the Gram-negative bacterial pathogens *P. aeruginosa* [47] and *Yersinia pestis* [29], and in the hypodermis to defend against the fungus *D. coniospora* [46]. We found that *C. elegans pmk-1(km25)* mutants were hypersusceptible to infection with *C. albicans* yeast (Figure 5A) and that PMK-1 was required for the basal and pathogen-induced expression of three antifungal immune effectors (*fipr-22/23*, *cnc-4* and *cnc-7*), but not *abf-2* (Figure 5B). The full spectrum of nematode sensitivity to *C. albicans* was not mediated by the genetic control of any of these four effectors because knockdown of each of these genes individually by RNA interference did not result in hypersusceptibility to fungal infection (data not shown). It is likely, however, that there is functional redundancy among immune effectors in *C. elegans*, as has been suggested previously [29], [32], [44], [49], [50]. That PMK-1 mediates resistance to *C. albicans* provides another line of evidence that yeast infection of the nematode stimulates host immune defenses. Moreover, the PMK-1-independent genetic regulation of the antifungal effector *abf-2* suggests that other pathways are also important in controlling the immune response toward *C. albicans*.

### The Host Response to *C. albicans* Involves Induction of Specific Defenses and Common Immune Genes

**Figure 6:**
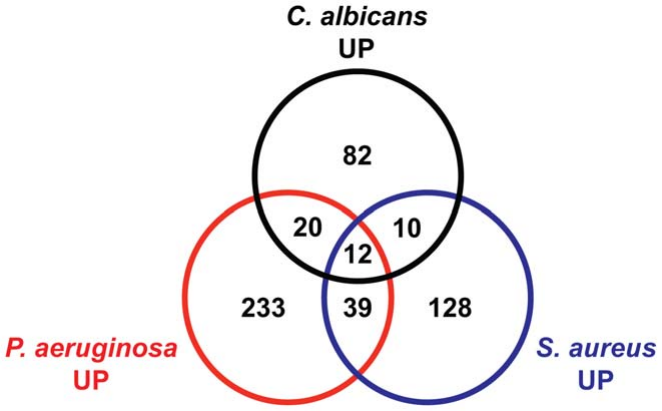
The transcriptional responses to *C. albicans* and bacteria comprise specific and overlapping gene sets. A Venn diagram illustrates the overlap of genes induced 2-fold or greater (*P*<0.01) by *C. albicans* (this study), *P. aeruginosa* [32] and *S. aureus* [34]. All microarrays were conducted using the Affymetrix platform. Animals were exposed to *C. albicans* and *P. aeruginosa* for 4 hours and to *S. aureus* for 8 hours. See Table S3A for gene identities.

To examine the specificity of the antifungal transcriptional response, we compared *C. albicans*-affected genes with those differentially regulated following infection with the bacterial pathogens *P. aeruginosa* [32] and *Staphylococcus aureus* [34] (*P*<0.01, >2-fold change) (Figure 6). The transcriptional responses induced by fungi, Gram-negative bacteria and Gram-positive bacteria overlapped only to a small extent and the majority of the *C. albicans*-affected genes were not involved in the response to *P. aeruginosa* or *S. aureus* (Figure 6, Table S3A). The *C. albicans*-specific genes in this comparison included the putative antifungal peptides *abf-2*, *fipr-22/23*, *cnc-7*, *thn-1* and the chitinases (*cht-1* and *T19H5.1*). We observed an overlap of 32 induced and 22 repressed genes between the transcriptional responses to *P. aeruginosa* and *C. albicans* (1.9 and 1.4 genes expected by chance alone, respectively; *P*<1.0×10^−16^ for both comparisons). Likewise, 22 upregulated and 25 downregulated genes were shared in the responses to *S. aureus* and *C. albicans* (2.8 and 2.2 genes expected by chance alone, respectively; *P*<1.0×10^−16^ for both comparisons). Interestingly, 12 genes were induced and 14 genes were repressed by all three pathogens. Despite the fact that the *C. albicans*-induced genes were determined using heat-killed *E. coli* as the control and the genes induced by *P. aeruginosa* and *S. aureus* were identified in separate studies that used live *E. coli* as the control, we detected an overlap of comparable significance between the transcriptional responses to these different organisms. 26% and 18% of *C. albicans*-induced genes were also upregulated by *P. aeruginosa* and *S. aureus*, respectively (Figure 6). Likewise, 17% of genes induced by *P. aeruginosa* four hours after infection were also upregulated by *S. aureus* and 11% of *S. aureus*-upregulated genes were induced by *M. nematophilum* [34]. Our data suggest that the nematode is able to specifically recognize *C. albicans* infection and mount a targeted response toward this fungus that involves antifungal defenses and a limited number of common core effectors.

### Both Heat-Killed and Live *C. albicans* Yeast Are Immunogenic to the Nematode

**Figure 7:**
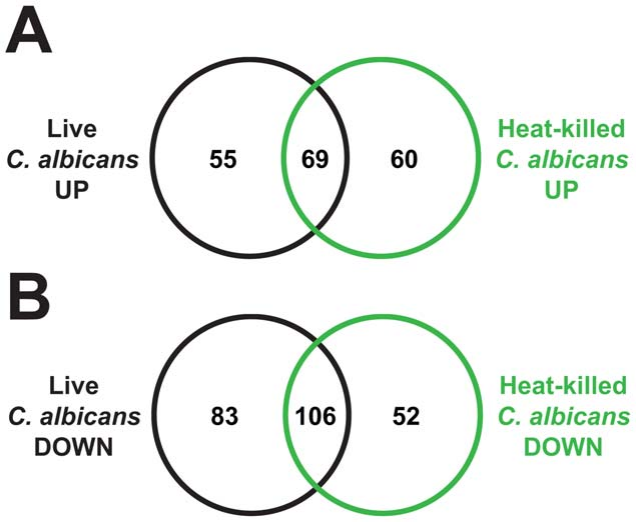
Heat-killed *C. albicans* yeast cells elicit a transcriptional response in *C. elegans* that overlaps with the response to live *C. albicans*. Venn diagrams give the overlap of *C. elegans* genes upregulated (A) and downregulated (B) at least 2-fold (*P*<0.01) in response to *C. albicans* and heat-killed *C. albicans*, each compared to heat-killed *E. coli*. See Table S3B for gene identities.

Components of the *C. albicans* cell wall, often referred to as PAMPs, are recognized by mammalian neutrophils, monocytes and macrophages [13], [15], [51]. In this study, we found that heat-killed *C. albicans* yeast accumulate within the *C. elegans* intestine (Figure 1B) and therefore postulated that the nematode transcriptional response to nonpathogenic, heat-killed fungi would reflect stimulation of host pathways by immunogenic components of the yeast cell wall. To explore the mechanisms of pathogen detection in the nematode, we fed animals heat-killed *C. albicans* as an additional condition in the transcriptome profiling experiment. Exposure to heat-killed *C. albicans* caused a transcriptional response in nematodes involving 287 genes (∼1.4% of the genome, *P*<0.01) (Table S1B). To determine whether these genes were also involved in defense against live *C. albicans* infection, we compared the genes differentially regulated by live and heat-killed *C. albicans* versus the baseline condition of heat-killed *E. coli*. Interestingly, there was significant overlap (69 genes, 56%) between genes induced by heat-killed *C. albicans* (vs. heat-killed *E. coli*) and live *C. albicans* (vs. heat-killed *E. coli*)(0.5 genes expected by chance alone, *P*<1.0×10^−16^)(Figure 7A, Table S3B). Likewise 106 of 189 genes (56%) repressed by *C. albicans* were also downregulated by heat-killed *C. albicans* (0.5 genes expected by chance alone, *P*<1.0×10^−16^)(Figure 7B, Table S3B). Interestingly, this overlap includes the majority of the most strongly regulated genes in both directions (Tables 1 and S1A).

These data constitute the first genome-wide analysis of the *C. elegans* transcriptional response to a heat-killed pathogen and afford several interesting observations. Heat-killed *C. albicans* yeast cells induce an antifungal transcriptional response in *C. elegans* despite being non-pathogenic (Figure 1). Genes upregulated by heat-killed *C. albicans* include several putative antifungal peptides (*abf-2*, *cnc-4*, *cnc-7*, *cht-1* and *thn-1*) and an abundance of secreted or intestinal expressed genes (Table 1), a profile similar to that of live *C. albicans*. Furthermore, heat-killed *C. albicans* caused the induction of core immune response genes. The comparison in Figure 6 showed that 42 genes were upregulated by *C. albicans* and either *P. aeruginosa* or *S. aureus*. Thirty-three genes (79%) in this set, including 7 out of 12 genes induced by all three pathogens, were also upregulated by heat-killed *C. albicans* (Table S3A). Together, these findings suggest that heat-killed *C. albicans* yeast induce host defenses and imply that a large part of the *C. elegans* transcriptional response may be mediated by detection of fungal PAMPs through Pattern Recognition Receptors, an evolutionarily-ancient system of pathogen sensing and signaling [52], [53].

Equally interesting, it seems that *C. elegans* also possesses mechanisms to respond directly to the virulence effects of *C. albicans*. We identified a smaller group of differentially regulated genes when we compared the transcriptome profiles from nematodes exposed to live *C. albicans* with those exposed to heat-killed *C. albicans*. The transcription of 62 genes (22 upregulated and 40 downregulated) changed in this analysis (*P*<0.01) (Table S1C) presumably in response to the pathogenicity of the fungus. 10 of the 22 genes (45%) upregulated by live *C. albicans* versus heat-killed *C. albicans* and 11 of the 40 downregulated genes (28%) were also differentially regulated by live *C. albicans* versus the baseline condition of heat-killed *E. coli* (0.12 and 0.36 genes respectively expected by chance alone, *P*<1.0×10^−16^ for both comparisons). These data are consistent with our observation that the induction of four putative antifungal effectors was reduced in the virulence-attenuated *C. albicans efg1Δ/efg1Δ cph1Δ/cph1Δ* double mutant strain compared to its isogenic, wild-type parent strain (Figure 4). Taken together, these data indicate that host recognition of *C. albicans* infection in the nematode involves at least two mechanisms: recognition of PAMPs and detection of factors associated with fungal virulence.

### Immune Specificity towards *C. albicans* Involves the Targeted Downregulation of Antibacterial Effectors

**Figure 8:**
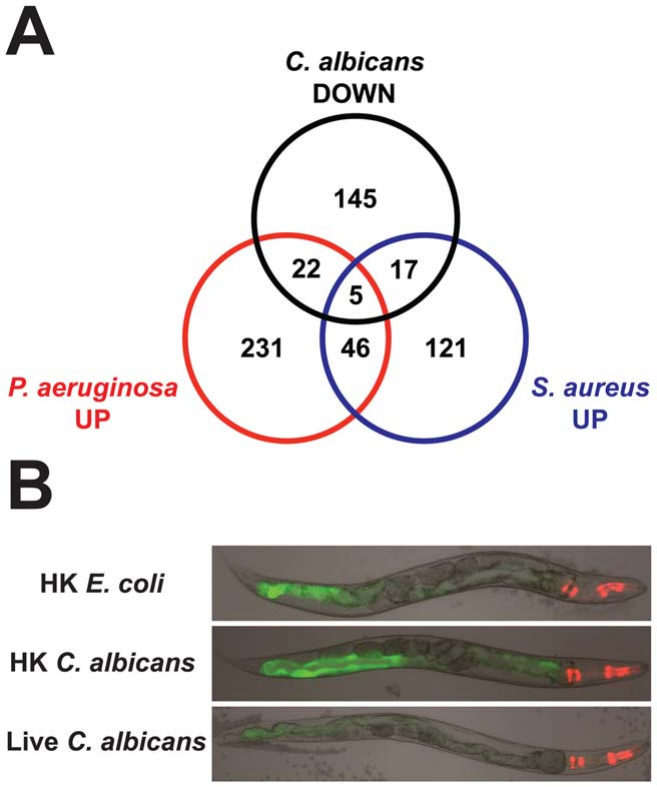
The *C. elegans* response to *C. albicans* involves the downregulation of antibacterial effectors. (A) A Venn diagram illustrates that a subset of *C. albicans* downregulated genes were upregulated after infection of *C. elegans* by pathogenic bacteria. See Table S3C for gene identities. (B) Transgenic *C. elegans* animals in which GFP expression was driven by the promoter for the C-type lectin *clec-60*, a secreted *S. aureus* immune effector that was downregulated by *C. albicans* in the microarray analysis, are shown. Worms were exposed to heat-killed (HK) *E. coli*, heat-killed *C. albicans* or live *C. albicans* for 20 hours at 25°C and then imaged. Green is *clec-60::GFP*. Red is the *myo-2::mCherry* co-injection marker used to identify transgenic animals.

Closer examination of the genes downregulated by *C. albicans* revealed an unexpected finding regarding antifungal immune specificity. We noticed that the most over-represented classes among the *C. albicans* downregulated genes (based on GO annotation) were involved in sugar or carbohydrate binding. Because these gene classes are upregulated in response to *P. aeruginosa* and *S. aureus* [32], [34], we postulated that some antibacterial defense effectors are specifically downregulated during infection with *C. albicans*. We therefore compared the 189 genes that are downregulated by *C. albicans* with the genes induced during infection with *P. aeruginosa* and *S. aureus*, and found a striking overlap (Figure 8A, Table S3C). Twenty-seven of the 189 downregulated *C. albicans* genes (14%) were induced by *P. aeruginosa*, which is 25-fold more than expected by chance alone (*P*<1.0×10^−16^). Likewise, 22 *S. aureus* response genes (12%) were downregulated by *C. albicans* (12-fold more than expected by chance alone, *P*<1.0×10^−16^). Thus, it seems that the nematode immune response to *C. albicans* involves the downregulation of a group of antibacterial defense genes.

We took two steps to confirm this observation. First, we used qRT-PCR to test the expression of seven genes differentially regulated by *C. albicans* and previously shown to be part of the *P. aeruginosa* transcriptional response (*irg-3*, *clec-67*, *K08D8.5*, *C17H12.8*, *F49F1.6*, *F35E12.5* and *F01D5.5*) [32]. All seven of these genes were strongly downregulated four hours after *C. albicans* infection (Table S2). We also assayed the expression of *clec-67*, *K08D8.5*, *C17H12.8* and *F49F1.6* 12 hours after infection and found that these genes continue to be transcriptionally repressed at this later time point (Figure S1). Two of these genes, *C17H12.8* and *F49F1.6*, were more strongly repressed at 12 hours compared to 4 hours after infection (*P*<0.01 and *P* = 0.07, respectively). As a second approach, we studied transgenic *C. elegans* animals in which the promoter for the *S. aureus* immune response gene *clec-60* was fused to GFP, allowing a visual readout of gene transcription. *clec-60* is a C-type lectin, a gene class important for nematode defense against bacterial pathogens [29], [32], [34], a member of which was shown to have direct antimicrobial activity in a mammalian system [54]. Consistent with the microarray analysis (Table S1A), we found that exposure to live *C. albicans* dramatically reduced GFP expression in *clec-60::GFP* transgenic animals compared to the basal expression of this gene on heat-killed *E. coli* (Figure 8B).

One interpretation of these data is that the downregulation of antibacterial effectors observed in the microarray analysis reflects the absence of bacteria in *C. albicans*-exposed animals rather than specific transcriptional repression of these genes during infection with pathogenic fungi. We therefore examined the genes that were downregulated in the comparison of live *C. albicans* versus heat-killed *C. albicans*, an experiment where bacterial antigens were not present in either condition. Of the 40 genes that were transcriptionally repressed in this comparison, 19 genes were also upregulated by *S. aureus* [34] or *P. aeruginosa* [32] (Table S1C)(0.08 genes expected by chance alone, *P*<1.0×10^−6^ for this comparison). For reasons that are not clear, only 6 of these 19 genes were also downregulated in the comparison of live *C. albicans* versus heat-killed *E. coli* (Table S3C); however, this overlap is significantly more than the 0.08 gene overlap expected by chance alone (*P* = 0.013). Therefore, we conclude that the nematode downregulates a group of antibacterial defense genes in response to some aspect of *C. albicans* virulence. It is also interesting that of the 44 antibacterial response genes shown in Figure 8 that were downregulated by *C. albicans*, 26 (59%) were also repressed by heat-killed *C. albicans* (Table S3C). Taken together, these data suggest that the nematode responds to components within heat-killed *C. albicans*, as well as factors associated with fungal virulence, to transcriptionally repress antibacterial immune responses.

One of the antibacterial genes downregulated in the comparison of live *C. albicans* and heat-killed *C. albicans* was *clec-60*. Thus, for additional confirmation of these data, we exposed *clec-60::GFP* transgenic animals to heat-killed *C. albicans*. As predicted from the microarray analysis, we found that expression of *clec-60::GFP* was visually unchanged compared to its basal level on heat-killed *E. coli* (Figure 8B). Furthermore, our finding that *C17H12.8* and *F49F1.6* were more strongly downregulated at 12 hours of infection (versus 4 hours)(Figure S1) suggests that the transcriptional repression of these antibacterial immune effectors is an active process associated with progression of fungal infection.

To understand the mechanism underlying the repression of antibacterial immune effectors during *C. albicans* infection, we assayed gene expression in *daf-16(mgDf47)* and *pmk-1(km25)* mutants. Troemel et al. previously showed that the p38 MAP kinase homolog PMK-1 controls the expression of many *P. aeruginosa* immune response genes [32]. In their analysis, they also observed that the FOXO/forkhead transcription factor DAF-16, a central regulator of nematode longevity, negatively regulates some *P. aeruginosa* defense genes, including a group of *pmk-1*-dependent genes. We therefore wondered whether DAF-16 negatively regulates antibacterial defense genes during infection with *C. albicans*. We determined the overlap of the *C. albicans* downregulated genes with the group of genes whose basal expression is negatively regulated by DAF-16 (so-called Class II genes from Murphy et al. [55]) and found a 24-gene overlap (more than the 2.6 genes expected by chance alone, *P*<1.0×10^−16^). From these analyses, we identified two genes (*clec-67* and *C17H12.8*) whose basal expression was previously reported as being induced by PMK-1 and negatively controlled by DAF-16 [32]. We examined the regulation of these genes during *C. albicans* infection and found that they were equally downregulated by *C. albicans* in both wild-type and *daf-16(mgDf47)* mutants (Figure S2), which suggests that DAF-16 is not responsible for this phenotype. In support of this observation, DAF-16::GFP remained localized to the cytoplasm following exposure to *C. albicans* and did not translocate into the nucleus, as it does when it is activated to regulate transcription (data not shown). We also wondered whether signaling through the PMK-1 pathway results in the downregulation of antibacterial immune effectors during *C. albicans* infection. However, the basal expression of *clec-67* and *C17H12.8* was profoundly affected by PMK-1 (Figure S2), which precluded analysis of differential regulation during *C. albicans* infection in *pmk-1(km25)* mutants. In summary, we show that antibacterial response genes are downregulated during *C. albicans* infection, including a group whose basal expression is repressed by DAF-16 and stimulated by PMK-1. We conclude that an unidentified mechanism, independent of DAF-16, accounts for this phenotype.

## Discussion

We show that the yeast form of *C. albicans* is pathogenic to the nematode and explore the mechanisms of immune activation by pathogenic fungi *in vivo*. Previous studies of *C. elegans* infection with bacterial pathogens have led to the characterization of a sophisticated and evolutionarily-conserved innate immune system in the nematode [21]. We found that the *C. elegans* is also able to specifically recognize and defend itself against *C. albicans*, the most common fungal pathogen of humans [1]. These data suggest that *C. elegans* integrates signals from *C. albicans* yeast and factors associated with its pathogenicity to mount a targeted defense response. We also found that nematode antifungal immunity involves the elaboration of immune effectors and the downregulation of antibacterial response genes.

### The *C. elegans* Immune Response to *C. albicans* is Mediated by the Detection of PAMPs and Fungal Virulence

Using a *C. elegans* pathogenesis assay that is conducted on solid agar plates, we show that *C. albicans* yeast cells kill worms in a manner dependent on live organisms and cause pathogenic distention of the nematode intestine during infection. Furthermore, we found that both heat-killed and virulence-attenuated *C. albicans* readily enter the nematode intestine, but are less pathogenic than wild-type yeast. While the mechanism of nematode mortality during *C. albicans* infection is unknown, these data suggest that some aspect of fungal virulence is required for yeast to infect and kill *C. elegans*.

In response to *C. albicans* attack, we found that the nematode mounts a pathogen-specific defense response that involves the induction of antifungal effectors and core immune genes. Interestingly, 56% of the genes involved in the transcriptional response to *C. albicans* infection were also differentially regulated by heat-killed *C. albicans*. These data suggest that a large part of the transcriptional response to *C. albicans* is elicited by fungal PAMPs. In mammals, heat-killed fungi also strongly activate host defenses and have been used to study PAMP-mediated immune signaling [13], [56]. In myeloid cells, cell wall components of heat-killed yeast (mannans and β-glucans) activate the pattern recognition receptors TLR2, TLR4, MR and dectin-1 to initiate antifungal immune responses [15]. Indeed, the process of heat killing may actually exaggerate innate immune responses in human cells by exposing fungal PAMPs. For example, β-glucans within the cell wall of *C. albicans* are normally covered by mannoproteins and thus blocked from detection by dectin-1 [13], [51]. Treatment of yeast cells with heat depletes this protective layer and exposes β-glucans, thereby enhancing dectin-1-mediated proinflammatory cytokine responses [56], [57].

The transcriptome profiling experiments and the expression analyses of nematodes infected with virulence-attenuated *C. albicans* suggest that factors associated with fungal virulence also elicit a transcriptional response in *C. elegans*. We do not know, however, whether these factors are derived from the host (e.g. as a consequence of cell damage) or from the pathogen. Recently, Moyes et al. found that human epithelial cells integrate inputs from *C. albicans* PAMPs via pattern recognition receptors together with “danger signals” perceived by the host during invasive fungal growth [14]. Interestingly, these researchers observed a biphasic activation of the p38 MAP kinase (MAPK) pathway, which was initially dependent on PAMP recognition and later on fungal burden and hyphal formation during invasive growth. We found a requirement for PMK-1, the nematode ortholog of the p38 MAP kinase, in the response to *C. albicans* infection. We therefore propose that similar mechanisms of pathogen detection involving the PMK-1 pathway exist in *C. elegans*. As in the human epithelium, the nematode may integrate signals from PAMPs together with inputs associated with fungal virulence to delineate a “pattern of pathogenesis [58]” specific to fungal infection. Further research is needed to determine the PAMPs that are detected by *C. elegans*, the intestinal pattern recognition receptors that bind them and the mechanisms by which fungal virulence is perceived in the nematode.

### Core Immune Effectors Are Activated by Bacterial and Fungal Pathogens

The immune response induced by Gram-negative bacteria, Gram-positive bacteria and fungi involve a small number of overlapping genes, a result that is somewhat surprising given the marked difference between prokaryotic and eukaryotic pathogens. Although others have also reported that the nematode mounts shared responses against different kinds of pathogens [34], [36], [59], our data are the first to define a core set of immune regulators involved in the defense against three prototypical nosocomial pathogens. These findings may ultimately have clinical implications. Our laboratories and others are using *C. elegans* pathogenesis assays as a means to identify novel antimicrobial therapies with immunomodulatory activity [60]. Thus, identifying compounds that boost these core immune response genes may yield novel therapies that can cure infection by three diverse, nosocomial pathogens and may be a strategy that can be applied in higher order hosts.

### Antibacterial Immune Effectors Are Downregulated by *C. albicans*

Unexpectedly, *C. albicans* infection of the nematode caused the downregulation of a number of antibacterial response genes including CUB-like genes, C-type lectins and ShK toxins. Moreover, it seems that both heat-killed (non-pathogenic) *C. albicans* and live (infectious) *C. albicans* can cause this repression. Interestingly, the basal expression of many of these genes is positively regulated by the p38 MAP kinase homolog PMK-1 and negatively regulated by DAF-16. How might the selective downregulation of these antibacterial response genes be evolutionarily advantageous for the worm? We know that the DAF-2 insulin/insulin-like growth factor receptor signals to the FOXO/forkhead transcription factor DAF-16 to control life span and stress resistance [61]–[63] and that DAF-16 negatively regulates *P. aeruginosa* immune response genes [32]. Troemel et al. postulated that immune response genes may be energetically expensive to make and thus their downregulation by DAF-16 under normal growth conditions may partially account for the lifespan-enhancing effects of DAF-2/DAF-16 pathway [32]. Irazoqui et al. found that the coordinated regulation of the immune response genes *clec-60/61* and *clec-70/71* influenced nematode survival. *C. elegans* animals carrying multiple copies of these gene clusters, which are induced during *S. aureus* infection, but not by *P. aeruginosa* or *C. albicans*, were more resistant to *S. aureus*, but were paradoxically hypersusceptible to *P. aeruginosa* [34]. We therefore propose that the transcriptional repression of antibacterial response genes, such as *clec-60* and *clec-70*, during *C. albicans* infection is an adaptive response. Given the recognized ability of FOXO/forkhead transcription factors to repress immune response genes both in *C. elegans* and in mammals [64], we hypothesized that DAF-16 activity would be responsible for this phenotype. However, our data suggest that an unidentified mechanism, independent of DAF-16, represses these genes following *C. albicans* infection.

We are not aware of other examples in metazoans in which activation of specific antimicrobial defenses results in the transcriptional downregulation of another immune response. In contrast, this phenomenon is well described in the immune response of *Arabidopsis thaliana*, a widely-studied, model laboratory plant [65]. In Arabidopsis, as well as other plants, two low molecular weight immune hormones, salicylic acid and jasmonic acid, are involved in the activation of distinct immune response pathways. Salicylic acid is primarily activated by obligate, biotrophic pathogens that require living plant cells to acquire nutrients. Jasmonic acid, on the other hand, is involved in the response to necrotrophic pathogens that kill host cells and then feed on the carcasses. In most cases, activation of salicylic acid-mediated signaling downregulates jasmonic acid signaling and vice versa. The mutual antagonism of the salicylic acid and jasmonic acid pathways is generally interpreted in terms of evolutionary tradeoffs between biotrophic and necrotrophic defenses [65]. Our data suggest that a similar antagonism may be occurring in *C. elegans* between bacterial and fungal defenses. That is, when confronted with a virulent fungal pathogen, *C. elegans* focuses its immune response on the production of specific antifungal effectors at the expense of antibacterial defenses. Our analysis of the genes downregulated by *P. aeruginosa* or *S. aureus* did not reveal a statistically significant overlap with the genes induced following exposure to *C. albicans*. An alternative explanation is that the genes that are downregulated by *C. albicans* actually encode key immune effectors important for defense against both bacterial and fungal pathogens. Instead of the host downregulating the expression of these genes, the transcriptional repression may reflect an offensive measure by *C. albicans* to enhance its ability to infect *C. elegans*.

### *C. elegans* Pathogenesis Assays Enable Analyses of *C. albicans* Virulence Mechanisms

In this study, we describe a novel *C. elegans* assay for the study of *C. albicans* yeast-mediated pathogenesis, which complements our hyphal formation model that we used to identify novel virulence determinants in *C. albicans* [33]. In our previous study, we screened a *C. albicans* mutant library containing homozygous mutations in 83 transcription factors [66] for clones attenuated both in their ability to form hyphae *in vivo* and kill *C. elegans* [33]. We uncovered several novel mediators of hyphal growth and showed that the *efg1Δ/efg1Δ cph1Δ/cph1Δ* double mutant [8], which is unable to program filamentation, was also attenuated for virulence in the *C. elegans* model, as it was in mammalian systems. The *efg1Δ/efg1Δ cph1Δ/cph1Δ* double mutant contain lesions in transcription factors that are the conserved readouts of the cAMP-mediated cascade (Efg1p) and the MAP-kinase cascade (Cph1p), each with well-described roles in the control of morphogeneis and virulence [8], [67]. In the current study, we show that this mutant was also attenuated for virulence in the *C. elegans* yeast-mediated pathogenesis assay. These data suggest that the *C. albicans* cAMP-mediated and MAP-kinase cascades also regulate yeast-specific virulence determinants and support the hypothesis that this morphogenic form is an important contributor to the pathogenic potential of wild-type fungi, as has been suggested by others [11], [68]–[70]. These data also indicate that the *C. elegans* system can be used in large-scale screens of *C. albicans* mutant libraries for novel virulence regulators possessed by yeast.

## Materials and Methods

### Strains and Media

*C. elegans* were maintained and propagated on *E. coli* OP50 as described [71]. The *C. elegans* strains used in this study were: N2 bristol [71], *pmk-1(km25)* [22], *daf-16(mgDf47)* [72], *fer-15(b26);fem-1(hc17)* [55], AU0157 [agEx39(*myo-2::cherry,clec-60::GFP*)] [28] and TJ356 [*zIs356* (*pDAF-16::DAF-16-GFP*;*rol-6*)] [73]. The *C. albicans* strains used in this study were DAY185 (*ura3Δ::λimm434/ura3Δ::λimm434 ARG4:URA3::arg4::hisG/arg4::hisG his1::hisG::pHIS/his1::hisG*) [74], SC5314 (clinical isolate) [75] and Can34 *(ura3Δ::λimm434/ura3Δ::λimm434 cph1Δ::hisG/cph1Δ::hisG efg1Δ::hisG/efg1Δ::hisG-URA3-hisG)* [8]. Unless otherwise specified, *C. albicans* DAY185 was used as the wild-type strain. Yeast strains were grown in liquid yeast extract-peptone-dextrose (YPD,BD) broth or on brain heart infusion agar containing 45 µg of kanamycin/ml at 30°C. Bacteria were grown in Luria Broth (LB, BD).

### *C. albicans*-*C. elegans* Solid Medium Pathogenesis Assay

The previously described protocol for pathogen infection of *C. elegans* was modified for these studies [76]. Freshly grown *C. albicans* of the indicated genotype were picked from a single colony and used to inoculate 1 mL of YPD broth, which was allowed to grow overnight with agitation at 30°C. The following day, 10 µL of yeast were spread into a square lawn in a 4 cm tissue culture plate (BD) containing 4 mL of BHI agar and kanamycin (45 µg/mL). For experiments that compared heat-killed and live *C. albicans*, cells were subjected to the exact same preparatory conditions. A single colony of yeast was grown in 1 mL BHI at 30°C overnight and then inoculated into 50 mL YPD. After approximately 20 hours of incubation, cells were split into two aliquots, collected by centrifugation and washed twice with sterile PBS (pH 7.4). One aliquot was resuspended in 1 mL PBS, exposed to 75°C for 60 minutes and washed again with sterile PBS. The other aliquot was processed in parallel with the heat-killed sample. Cells were suspended in 25 mL PBS, incubated at room temperature for 60 minutes and washed again with sterile PBS. 10 µL of this sample were added to the killing assay plates. To heat kill *E. coli*, a similar protocol was followed except that a single colony was inoculated into 50 mL LB and allowed to grow overnight at 37°C. Cells were exposed to 75°C for 30 minutes. In both cases, heat-killed organisms were plated on YPD or LB agar to ensure no viable organisms remained. 50 µL of heat-killed cells were added to the assay plates. The plates were then incubated for approximately 20 hours at 30°C. The next day, a Pasteur pipette molded into the shape of hockey stick was used to gently scrape excess yeast off the top of the thick *C. albicans* lawn. This step greatly facilitated scoring the animals as live or dead on subsequent days and did not affect the pathogenicity of *C. albicans* (data not shown). Five-fluoro-2′-deoxyuridine (FUDR; 75–100 µg/mL) was added to the plates 1 to 2 hours before the start of the assay to reduce the growth of progeny and prevent matricidal killing of nematodes by *C. albicans*. Thirty to forty young adult animals of the indicated genotype were added to each of three assay plates per condition studied. Although it is possible that microorganism inocula varied among individual worms, we doubt that such variation affected the pathogenicity of *C. albicans* in our assay since we observed similar killing kinetics in replicate experiments. Animals were scored as live or dead on a daily basis by gently touching them with a platinum wire. Worms that crawled onto the wall of the tissue culture plate were eliminated from the analysis. All killing assays were conducted at 25°C. *C. elegans* survival was examined using the Kaplan-Meier method and differences were determined with the log-rank test (STATA 6; STATA, College Station, TX).

### Microarray Analysis of *C. albicans* Infected Nematodes

N2 animals were synchronized by hypochlorite treatment. Arrested L1s were plated on 10 cm NGM plates seeded with *E. coli* OP50 and grown at 20°C until they were young adults. Animals were then added to 10 cm plates containing 20 mL of BHI agar (with 45 µg of kanamycin/ml) and live *C. albicans*, heat-killed *C. albicans* or heat-killed *E. coli*. Plates were prepared using the method described above except 50 µL of cells were added to the plates for each condition together with 200 µL of PBS to facilitate even dispersion of the microbes. Three separate biological replicates of nematodes were exposed to these conditions for 4 hours at 25°C. RNA was extracted using TRI Reagent (Molecular Research Center) according to the manufacturer's instructions and purified using an RNeasy column (Qiagen). RNA samples were prepared and hybridized to Affymetrix full-genome GeneChips for *C. elegans* at the Harvard Medical School Biopolymer Facility following previously described protocols [32] and instructions from Affymetrix. Data were analyzed using Resolver Gene Expression Data Analysis System, version 5.1 (Rosetta Inpharmatics). Three biologic replicates per condition were normalized using the Resolver intensity error model for single color chips [77]. Conditions were compared using Resolver to determine the fold change between conditions for each probe set and to generate a *P* value using a modified *t*-test. Probe sets were considered differentially expressed if the fold change was 2-fold or greater (*P*<0.01). When comparing datasets, the overlap expected by chance alone was determined in 50 groups of randomly selected *C. elegans* genes using Regulatory Sequence Analysis Tools (http://rsat.ulb.ac.be/rsat/), a technique that has been used for similar analyses [78]. *P* values were determined using chi-square tests. Analyses for over-representation of GO annotation categories were performed using DAVID Bioinformatics Resources 6.7 from the National Institute of Allergy and Infectious Diseases [79], [80]. Two databases were used to determine the expression patterns for selected genes: Expression Patterns for *C. elegans* Promoter::GFP Fusions (http://gfpweb.aecom.yu.edu/) [81] and NEXTDB [82].

### Quantitative RT-PCR (qRT-PCR) Analyses

Animals were treated and RNA was extracted as described above. RNA was reverse transcribed to cDNA using the Retroscript kit (Ambion). cDNA was analyzed by qRT-PCR using a CFX1000 machine (Bio-Rad) and previously published primers [32], [39], [41]. Primer sequences for *fipr-22/23* (GCTGAAGCTCCACACATCC and TATCCCATTCCTCCGTATCC) and *cnc-7* (CAGGTTCAATGCAGTATGGCTATGG and GGACGGTACATTCCCATACC) were designed for this study, checked for specificity against the *C. elegans* genome and tested for efficiency with a dilution series of template. The primer set for *fipr-22/23* cannot distinguish between these two genes owing to sequence similarity. All values were normalized against the control gene *snb-1*, which has been used previously in qRT-PCR studies of *C. elegans* innate immunity [31], [32], [48], [83]. Analysis of the microarray expression data revealed that the expression of *snb-1* did not vary under the conditions tested in our experiment. Fold change was calculated using the Pfaffl method [84] and compared using *t*-tests.

### Microscopy

Nematodes were mounted onto agar pads, paralyzed with 10 mM levamisole (Sigma) and photographed using a Zeiss AXIO Imager Z1 microscope with a Zeiss AxioCam HRm camera and Axiovision 4.6 (Zeiss) software.

### Accession Numbers

Accession numbers for the genes and gene products mentioned in this paper are given for Wormbase, a publically available database that can be accessed at http://www.wormbase.org. These accession numbers are *pmk-1 (B0218.3)*, *abf-2 (C50F2.10)*, *fipr-22/23 (C37A5.2/4)*, *cnc-4 (F09B5.9)*, *cnc-7 (F53H2.2)*, *cht-1 (C04F6.3)*, *T19H5.1*, *irg-3 (F53E10.4)*, *clec-67 (F56D6.2)*, *K08D8.5*, *C17H12.8*, *F49F1.6*, *F35E12.5*, *F01D5.5*, *clec-60 (ZK666.6)* and *daf-16 (R13H8.1)*. The microarray dataset can be downloaded from the National Center for Biotechnology Gene Expression Omnibus (GEO; http://www.ncbi.nlm.nih.gov/geo). The accession number for these data is GSE2740.

## Supporting Information

Figure S1 **The transcriptional responses to** ***C. albicans*** **are dynamic during infection.** qRT-PCR analysis of wild-type nematodes 4 and 12 hours after infection reveals that *abf-2* is more strongly induced (*P*<0.01) and *fipr-22/23* expression is statistically unchanged. *cnc-4* and *cnc-7* return to baseline expression levels at 12 hours after infection. The antibacterial response genes *C17H12.8* and *F49F1.6* were more strongly downregulated at the later time point (*P*<0.01 and *P* = 0.07, respectively). Expression of *K08D8.5* was unchanged and *clec-67* became less strongly downregulated. Data are the average of three biological replicates (4 hour time point) or two biological replications, each measured in duplicate (12 hour time point). Error bars represent SEM. If error bars are not visible, the variation is smaller than the point on the graph.
(TIF)

Figure S2 **Downregulation of antibacterial response genes by** ***C. albicans*** **is not dependent on the FOXO/Forkhead Transcription Factor DAF-16.** Wild-type (N2) and *pmk-1(km25)* [left side] and N2 and *daf-16(mgDf47)* [right side] young adult animals were exposed to the indicated food source and the transcription levels of the indicated genes were determined using qRT-PCR. Expression is relative to wild-type on heat-killed *E. coli* and the data are presented as the average of two biological replicates, each conducted in duplicate and normalized to a control gene with error bars representing SEM.
(TIF)

Table S1 **Differentially expressed genes in the microarray experiments.** Presented are the lists of Affymetrix probe sets whose expression changed more than 2-fold (*P*<0.01) in the following exposure comparisons: live *C. albicans* versus heat-killed *E. coli* (A), heat-killed *C. albicans* versus heat-killed *E. coli* (B), live *C. albicans* versus heat-killed *C. albicans* (C). In A, the genes that were also differentially regulated in B and C are given in blue and red, respectively. In C, the genes in this list that were also upregulated by *S. aureus*, *P. aeruginosa* or both pathogens are annotated in the far right column. If two probe sets correspond to the same gene and both are differentially regulated in the array, then one is given in *italics*. If one probe set recognizes more than one gene, each gene is listed as a separate entry. A summary of the data is presented at the bottom of each worksheet.
(XLS)

Table S2 **Correlation between the microarray data and qRT-PCR analyses.** The fold change for the indicated *C. elegans* genes was determined four hours after exposure to the laboratory reference strain *C. albicans* DAY185 versus heat-killed *E. coli* in the microarray analysis and from qRT-PCR analyses of RNA set A and B. RNA set A was from the three biological replicates that were used in the microarray analysis. RNA set B was from three independent replicates. The fold change for 8 of these genes was also determined following a four-hour exposure to the *C. albicans* clinical isolate SC5314 versus heat-killed *E. coli*. The table gives the average fold change from three biological replicates, each normalized to a control gene (biological replicates of the SC5314 data were also tested in duplicate). 95% confidence intervals for the qRT-PCR data are given in parentheses. n.t. equals “not tested.”
(DOC)

Table S3 **A. Shared transcriptional signature between** ***C. albicans*** **,** ***P. aeruginosa*** **and** ***S. aureus*** **.** Genes that were induced or repressed by all three pathogens, by *C. albicans* and *P. aeruginosa* and by *C. albicans* and *S. aureus* at least 2-fold (*P*<0.01) are presented (see Figure 6). **B. Presumptive** ***C. albicans*** **PAMP-response genes.** The genes that were upregulated and downregulated at least 2-fold (*P*<0.01) by both heat-killed and live *C. albicans* (versus heat-killed *E. coli*) are listed (see Figure 7). **C. Antibacterial genes are repressed during** ***C. albicans*** **infection.** Listed are the genes that are repressed by *C. albicans* at least 2-fold (*P*<0.01) and induced by both *P. aeruginosa* and *S. aureus*, just *P. aeruginosa* or just *S. aureus* (see Figure 8). Additional columns in A and C indicate whether the gene was activated (or repressed) by heat-killed *C. albicans* (versus heat-killed *E. coli*) or by live *C. albicans* (versus heat-killed *C. albicans*). “-” indicates that expression was not affected.
(XLS)
