# Supplementary figures and images for: Candida albicans Infection of Caenorhabditis elegans Induces Antifungal Immune Defenses
Source: PLoS Pathog. 2011 Jun 23;7(6):e1002074. doi: 10.1371/journal.ppat.1002074 (PMC3121877; doi:10.1371/journal.ppat.1002074)

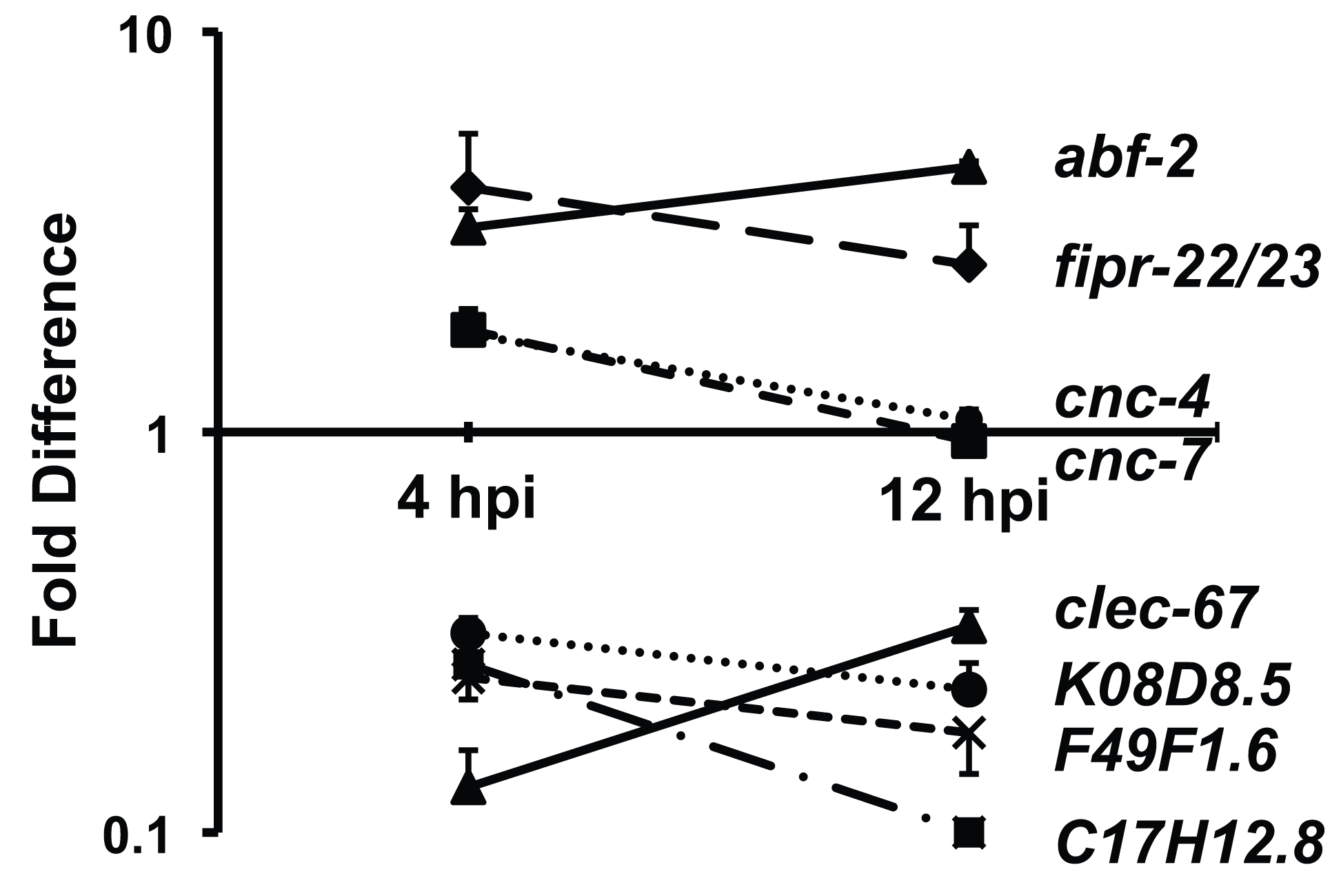

Supplement: Figure S1 — The transcriptional responses to C. albicans are dynamic during infection. qRT-PCR analysis of wild-type nematodes 4 and 12 hours after infection reveals that abf-2 is more strongly induced (P<0.01) and fipr-22/23 expression is statistically unchanged. cnc-4 and cnc-7 return to baseline expression levels at 12 hours after infection. The antibacterial response genes C17H12.8 and F49F1.6 were more strongly downregulated at the later time point (P<0.01 and P = 0.07, respectively). Expression of K08D8.5 was unchanged and clec-67 became less strongly downregulated. Data are the average of three biological replicates (4 hour time point) or two biological replications, each measured in duplicate (12 hour time point). Error bars represent SEM. If error bars are not visible, the variation is smaller than the point on the graph. (TIF) [file ppat.1002074.s001.tif]

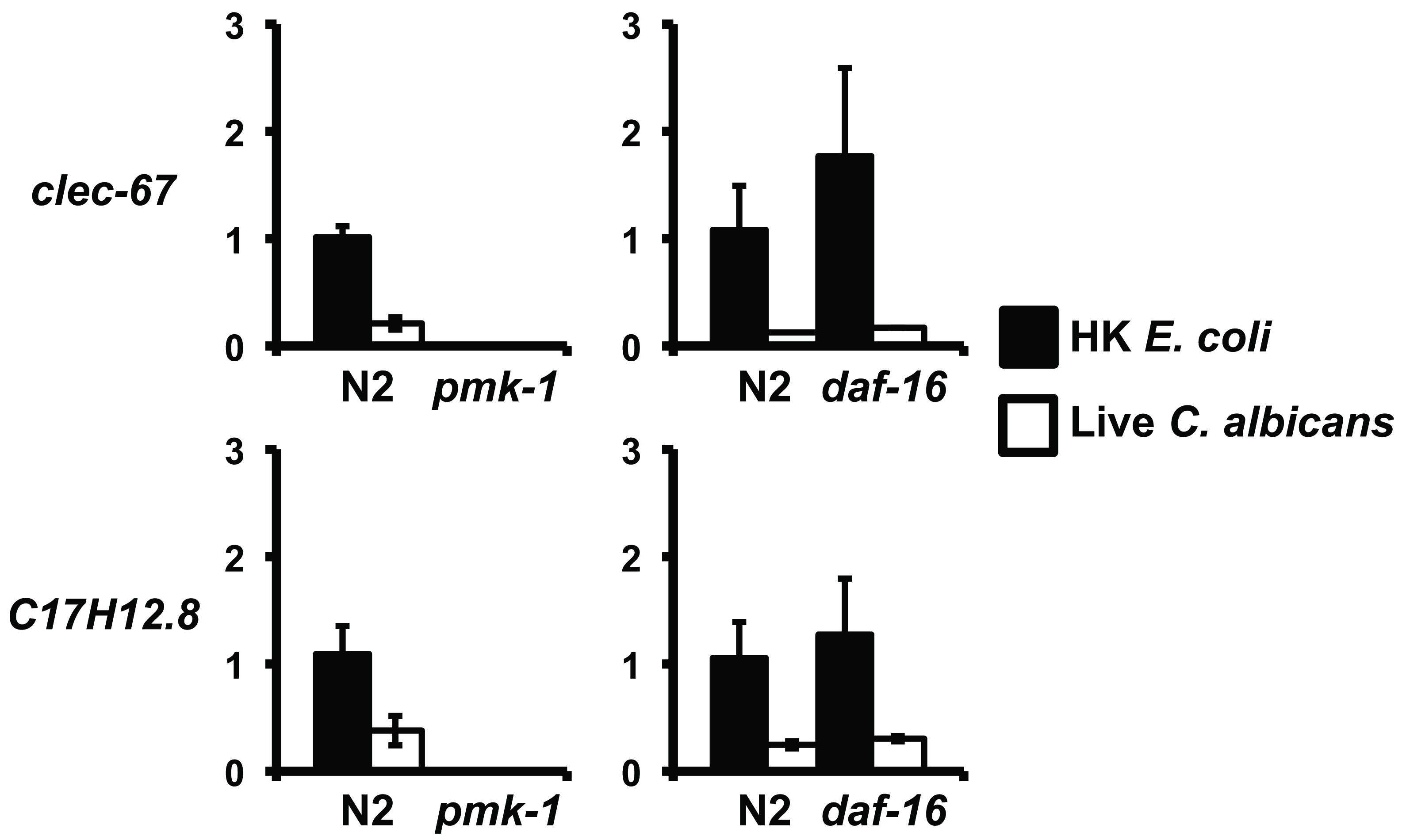

Supplement: Figure S2 — Downregulation of antibacterial response genes by C. albicans is not dependent on the FOXO/Forkhead Transcription Factor DAF-16. Wild-type (N2) and pmk-1(km25) [left side] and N2 and daf-16(mgDf47) [right side] young adult animals were exposed to the indicated food source and the transcription levels of the indicated genes were determined using qRT-PCR. Expression is relative to wild-type on heat-killed E. coli and the data are presented as the average of two biological replicates, each conducted in duplicate and normalized to a control gene with error bars representing SEM. (TIF) [file ppat.1002074.s002.tif]
