# Supplementary material for: Candida albicans Infection of Caenorhabditis elegans Induces Antifungal Immune Defenses
Source: PLoS Pathog. 2011 Jun 23;7(6):e1002074. doi: 10.1371/journal.ppat.1002074 (PMC3121877; doi:10.1371/journal.ppat.1002074)
Supplement: Table S2 — Correlation between the microarray data and qRT-PCR analyses. The fold change for the indicated C. elegans genes was determined four hours after exposure to the laboratory reference strain C. albicans DAY185 versus heat-killed E. coli in the microarray analysis and from qRT-PCR analyses of RNA set A and B. RNA set A was from the three biological replicates that were used in the microarray analysis. RNA set B was from three independent replicates. The fold change for 8 of these genes was also determined following a four-hour exposure to the C. albicans clinical isolate SC5314 versus heat-killed E. coli. The table gives the average fold change from three biological replicates, each normalized to a control gene (biological replicates of the SC5314 data were also tested in duplicate). 95% confidence intervals for the qRT-PCR data are given in parentheses. n.t. equals “not tested.” (DOC) [file ppat.1002074.s004.doc]

| **Gene name** | **Microarray** | **RNA set A** | **RNA set B** | **SC5314** |
| --- | --- | --- | --- | --- |
| *abf-2* | 5.9 | 4.9(±2.0) | 3.2(±0.7) | 4.5(±1.5) |
| *fipr-22/23* | 6.7 | 3.6(±0.8) | 4.1(±3.5) | 6.0(±1.4) |
| *cnc-4* | 4.1 | 2.3(±1.0) | 1.8(±0.3) | 4.4(±2.4) |
| *cnc-7* | 2.9 | 1.8(±0.7) | 1.8(±0.5) | 2.3(±0.7) |
| *F35E12.5* | 3.5 | 4.1(±1.4) | 0.3(±0.1) | n.t. |
| *irg-3* | 0.2 | 0.4(±0.1) | 0.5(±0.2) | n.t |
| *clec-67* | 0.2 | 0.2(±0.1) | 0.1(±0.06) | 0.2(±0.08) |
| *K08D8.5* | 0.4 | 0.3(±0.1) | 0.2(±0.1) | 0.2(±0.1) |
| *C17H12.8* | 0.5 | 0.4(±0.1) | 0.3(±0.1) | 0.3(±0.1) |
| *F49F1.6* | 0.3 | 0.4(±0.2) | 0.3(±0.06) | 0.3(±0.2) |
| *F01D5.5* | 0.2 | 0.3(±0.05) | 0.3(±0.04) | n.t. |
